# Cryopreservation of the gorgonian endosymbiont *Symbiodinium*

**DOI:** 10.1038/srep18816

**Published:** 2016-01-12

**Authors:** Gabriella Chong, Sujune Tsai, Li-Hsueh Wang, Chih-Yang Huang, Chiahsin Lin

**Affiliations:** 1 National museum of Marine Biology & Aquarium, 2 Houwan Road, Checheng, Pingtung, 944, Taiwan; 2 Institute of Marine Biology, National Dong Hwa University, 2 Houwan Road, Checheng, Pingtung, 944, Taiwan; 3 Department of Biotechnology, Mingdao University, 369 Wen-Hua Road, Peetow, ChangHua, 52345, Taiwan; 4 Department of Post Modern Agriculture, Mingdao University, 369 Wen-Hua Road, Peetow, Chang Hua, 52345, Taiwan; 5 Department of Aquaculture, National Taiwan Ocean University, 2 Beining Road, Jhongjheng, Keelung, Taiwan

## Abstract

The study focused on finding a suitable cryoprotectant (CPA) and an optimum freezing protocol for the cryopreservation of the endosymbiotic dinoflagellates (*Symbiodinium*, clade G) of *Junceella fragilis* wherein the success of experiments is crucial to both scientific and ecology studies. A two-step freezing technique was developed. The viability of the thawed dinoflagellates was assayed using the adenosine triphosphate (ATP) bioassay for the first time and was further confirmed through the culturing of dinoflagellates *in vitro*. The results suggested that 30 min was the most suitable holding time for the dinoflagellates, and the samples produced highest viability when suspended at 5 cm from the surface of LN_2_. Results also showed that 1 M methanol with 0.4 M sucrose was the most effective CPA, yielding the highest viability (56.93%). Although cell densities of both cryopreserved and control group suffered an initial decline of culture, the cell densities were maintained throughout the remaining duration. In the present study, the cryopreservation of clade G endosymbiont algae was studied for the first time and the method described here could be applied for future studies on symbiotic algae cryopreservation.

Coral reefs play a vital role in maintaining a balanced ecosystem by acting as natural breakwaters and sequestering carbon. Despite their known importance, coral populations continue to suffer degradation from excessive coastal development and unfavourable climate change^1^. The Global Coral Reef Monitoring Network^2^ has estimated that the world has lost 19% of its original reefs. In Taiwan, results from reef check showed that more that 80% of the 80 reef sites are under stressful conditions^3^. It has also been estimated that by 2030, 60% of the global reef area may be lost. This has led to the urgent need for a direct, fast and efficient effort for the conservation of reefs and corals. In pristine reefs, corals maintained their survivability by establishing symbiotic relationships with marine dinoflagellates; dinoflagellates supply nutrients and energy to the host coral and the relationship is often obligatory and crucial for the survival of the coral^4^. *Dinoflagellate* will be used hereafter to refer to coral-isolated symbiotic algae. Cryopreservation has been extensively applied to cells and tissues because of its cost effectiveness, easy maintenance, ability to maintain cellular viability for prolonged periods and ultimately, its ability to maintain genetic diversity among the population that was cryopreserved^5^. Hence, for the success of future coral propagation, the importance of cryopreservation of the symbiotic dinoflagellates should not be overlooked.

Despite limited information regarding the physiology of symbiotic dinoflagellates, the successful cryopreservation of symbiotic dinoflagellates has been reported. Santiago-Vazquez *et al.*^6^, cryopreserved symbiotic dinoflagellate of the octocoral *Pseudopterogorgia elisabethae* using ethanol and methanol as the cryoprotectants (CPAs). The dinoflagellates were successfully cultured after thawing. The two-step freezing is the most common technique for algae cryopreservation^7^. It starts with a period of equilibration of the CPA with the algae sample. After samples have undergone vapour cooling to a designated temperature using liquid nitrogen (LN_2_), the samples can be plunged into LN_2_ and stored at −196 °C^8^. Because not every species can be cryopreserved with the same cryopreservation protocol^9^, each criteria in the protocol such as CPAs, equilibration time, freezing rate and thawing temperature should be species-specific for effective preservation.

Although many studies have been conducted in the field of algal cryopreservation, standardised viability assays for post-thawed algae have not been established. In past studies, various viability tests were used such as staining^6^,^10^, metabolic viability^6^,^11^ and assessing cell density for post-thaw algae cultured *in vivo*. These methods could yield discrepancies in results; thus, cryopreservation techniques were sometimes incomparable^12^. Nevertheless, most authors have used a combination of viability assays for their studies. Cellular metabolic bioassays, such as ATP bioassay, have been proven to be effective for assessing post-thaw coral oocytes and sperm sacs by Tsai *et al.*^13^,^14^. However, no study has been conducted using the ATP bioassay to assess the cryopreservation of dinoflagellates.

The present study aimed to establish a cryopreservation protocol for the dinoflagellates of *J. fragilis* by determining the optimum freezing parameters, including the sample distance from the LN_2_ surface and the equilibration time, and the type of CPA used. Last, dinoflagellates will be cultured for the further assessment of viability.

## Results

### Cooling Temperature

Table 1 shows the final temperatures of the samples that were placed at different distances from the surface of LN_2_. Samples nearest to LN_2_ (3 cm) had the highest temperature change (−196.0 °C) with the lowest final temperature at −172.2 °C, whereas the sample placed furthest from the surface of LN_2_ (7 cm) had the least temperature change (−136.9 °C) with the highest final temperature at −112.9 °C. The amount of time required for samples to reach their final temperatures differed in a non-linear fashion; both samples that were placed at 5 cm and 7 cm from LN_2_ achieved their final temperatures in a shorter period of time compared with samples that were placed at 3 cm from LN_2_. This suggested that the cooling process was non-linear and there may be different cooling mechanisms and curves at different distances for each sample. For example, some samples may experience a faster initial cooling rate and gradually slowed down, whereas others may experience a slower initial cooling rate and gradually increased in the rate of cooling with time.

### Effects of holding time and distance to LN_2_ surface

**Figure 1:**
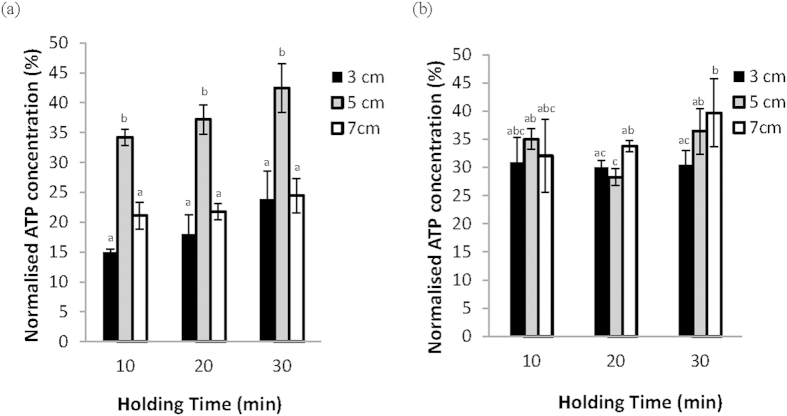
Intracellular ATP concentrations of dinoflagellates with 3 M MeOH. (**a**) and 2 M DMSO (**b**) for 10, 20 and 30 min holding time at different heights above the surface of LN_2_. Error bars represent standard error of the mean, and different letters represent significant differences (p < 0.05) between all combinations of holding times and distances from the LN_2_ interface.

The effect of holding time (10, 20 and 30 min) on the viability of algae was examined. The results showed that when samples were preserved with either methanol (Fig. 1a) or DMSO (Fig. 1b) and suspended at 3, 5, or 7 cm from the LN_2_ interface, a 30-minute hold time at a 5-cm distance was found to yield the highest normalized ATP concentration when methanol was used as a cryoprotectant, although there was not a significant effect of hold time on this distance (Fig. 1a). Within the 30 min holding time category, the optimum distance to LN_2_ surface was selected. When cryopreserved with 3 M methanol, 5 cm with the 30 min of holding time showed the highest viability with statistical significance (p < 0.05). For cryopreservation with 2 M DMSO, 7 cm with the 30 min of holding time gave the highest viability (p > 0.05). Nevertheless, there was no statistically significant difference between the 5 cm and 7 cm distances when DMSO was added as CPA (Fig. 1b). Hence, a holding time of 30 min with a suspension distance of 5 cm from LN_2 _was used in subsequent experiments.

### Effects of Cryoprotectants on algae

**Figure 2:**
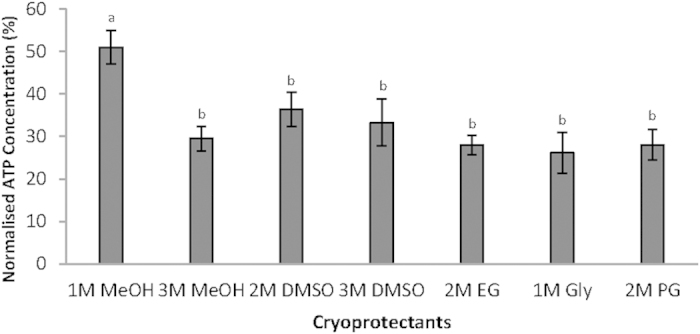
Effect of various cryoprotectants on intracellular ATP concentrations. Error bars represent standard error of the mean, and different letters represent significant differences (p < 0.05) between all combinations of cryoprotectants.

CPAs were added for protecting the samples during the transition to extreme low temperatures. Of the seven CPAs examined, 1 M MeOH (Fig. 2) yielded the highest post-thaw ATP concentration (50.99%), followed by 2 M DMSO (36.42%) (Fig. 2). The remaining five CPAs yielded intermediate results: 3 M DMSO, 33.29%; 3 M MeOH, 29.53%; 2 M PG, 28.05%; 2 M EG, 27.94% and 1 M Gly, 26.16%.

### Sucrose treatment

**Figure 3:**
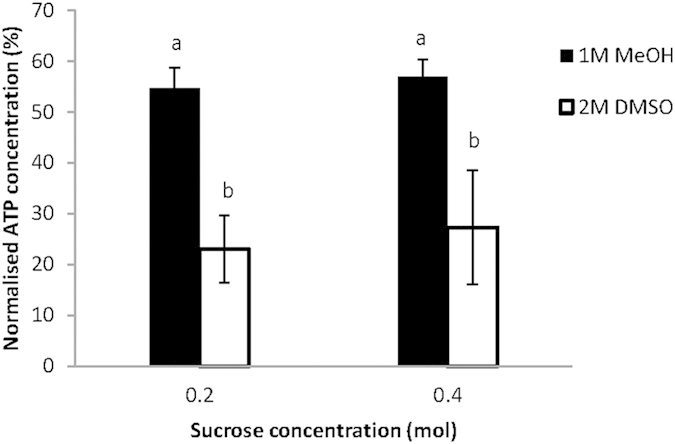
Cryopreservation of dinoflagellate using MeOH and DMSO with sucrose. Error bars represent standard error of the mean, and different letters represent significant differences (p < 0.05) between all combinations of cryoprotectants and sucrose concentration.

Both 1 M MeOH and 2 M DMSO were tested with sucrose for examining the effects of combined CPAs on post-thaw viability of dinoflagellate. As shown in Fig. 3, the addition of 0.2 M and 0.4 M sucrose in 1 M MeOH increased the viability from 50.99% to 54.60% and 56.93%, respectively. As for the 2 M DMSO, the addition of sucrose at either 0.2 M or 0.4 M was found to have a negative effect as the viability decreased from 36.42% to 23.01% and 27.29%, respectively. It was also noted that the effect of sucrose was influenced by the type of CPA used. Sucrose in both concentrations demonstrated a higher protection on the cryopreservation of algae when combined with MeOH.

### Cell Density

**Figure 4:**
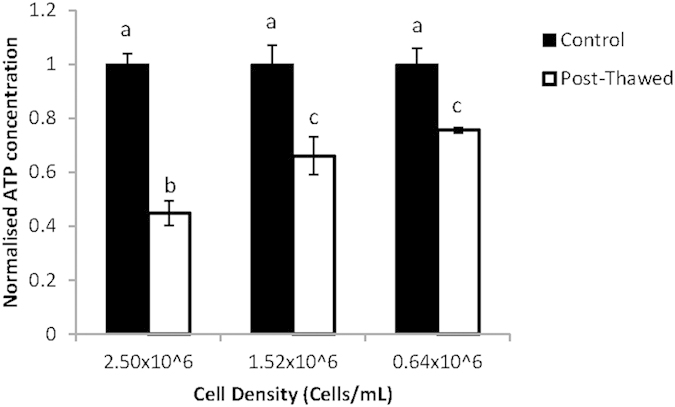
Viability of dinoflagellates when cryopreserve in different concentration. Error bars represent standard error of the mean, and different letters represent significant differences (p < 0.05) between all combinations of cell densities.

In the cell density experiment, three densities were tested that differed by approximately 1 × 10^6 ^cells/mL intervals. The cells here were approximated number of cells within the sample. The experiment indicated that a lower cell density (0.64 × 10^6 ^cells/mL) contributed to a significantly higher post-thaw viability when compared with a higher cell density (2.50 × 10^6 ^cells/mL; Fig. 4), but is not significantly higher when compared to the intermediate concentration of 1.5 × 10^6 ^cells/mL.

### Algae Culture

**Figure 5:**
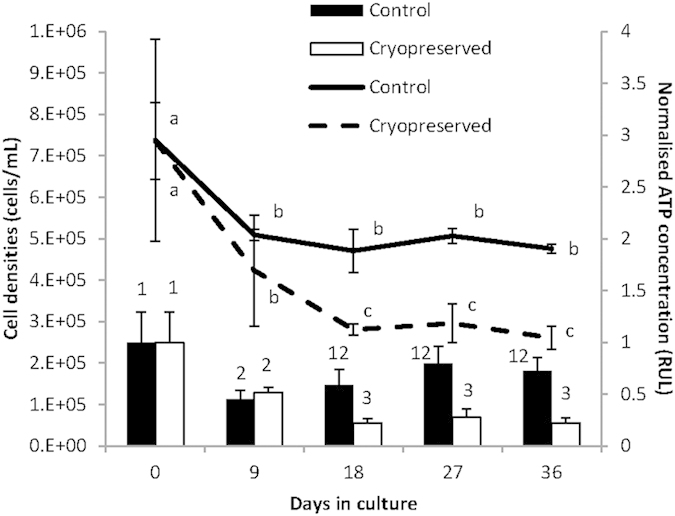
Cell density (lines and left y-axis) and normalised ATP concentration (columns and right y-axis; an indicator of viability) of control and cryopreserved *Symbiodinium* isolated from *J. fragilis* and cultured for 36 days. The letters and numbers over the lines and columns respectively, indicate differences (p < 0.05) between treatments and over time for the cell density and ATP concentration data, respectively.

After cryopreservation (30 min holding time, 5 cm distance above the surface of LN_2_, 1 M MeOH with 0.4 M sucrose), algae were thawed and incubated for a period of 36 days. It was observed that the cell density of the control (freshly-isolated *Symbiodinium*) and cryopreserved algae suffered a significant decrease in the first week of incubation (Fig. 5). After the initial decrease from 7 × 10^5^ to 5 × 10^5^, cell density of the control group remained stable throughout the remaining days. However, the cell number of the cryopreserved group continued to decrease until 18 days and remained at the same level throughout the remaining 36 days. ATP concentration in algae either control or cryopreserved groups decreased gradually with increasing culture period of 9 days and then ATP concentration plateaued after 18 days in culture. In control groups, although ATP concentration in algae decreased significantly after 9 days of culture, ATP concentrations were significantly higher than cryopreserved algae after culture for the remaining 36 days (Fig. 5).

## Discussion

Various studies on cryopreservation of algae indicated different preferences for the use of CPAs. Thus far only Santiago-Vazquez *et al.*^6^, reported on the cryopreservation of coral-isolated symbiotic algae. Through genetic sequencing, our results correlated with van Oppen *et al.*^15^ which identified symbiotic algae isolated from *J. fragilis* to be primarily clade G *Symbiodinium*. During the cooling procedure, samples that were suspended closer to the surface of LN_2_ had the lowest final temperatures than those suspended further from the surface of LN_2_. The final temperatures depending on the distance from the surface of LN_2_ affect the rate of cooling for the cryopreserved samples. According to Hori *et al.*^16^, samples placed furthest from the LN_2_ experienced slower cooling rates. However, this phenomenon was not observed in our experiments as samples at 5 or 7 cm from the surface of the LN_2_ recorded faster cooling rate than those at 3 cm. This suggested that cooling rate during the two-step freezing process differs from the control-cooling method which exhibited a linear cooling curve. Our results indicated that the two-step cooling exhibited non-linear cooling rates, and the samples at different distances from the surface of LN_2_ cooled with different cooling patterns.

Preliminary toxicity tests had been carried out at room temperature by incubating various CPAs at different concentrations with the isolated symbiotic algae from *J. fragilis*. Thus, the CPAs and the concentrations that were used here had already been found to be less harmful (unpublished results). In the present study, MeOH was more effective than DMSO for the cryopreservation of symbiotic algae. Furthermore, 1 M MeOH combined with 0.4 M sucrose gave the best results. The use of MeOH is agreeable with the results from Santiago-Vázquez^6^ wherein 20% MeOH successfully cryopreserved *Symbiodinium* clade B isolated from *P. elisabethae*. However, our results were in contrast to those of Joseph *et al.*^17^ where 10% glycerol produced the highest post-thaw viability (5%–40%) in marine algae. In the present study, 1 M glycerol was not effective because the post-thaw viability was the lowest (26.16%) among the seven CPAs tested.

According to Taylor and Fletcher^18^, MeOH, DMSO and glycerol are the common CPAs for the cryopreservation of algae. Both MeOH and DMSO are permeating CPAs; they penetrate into the cell to replace the water, preventing osmotic imbalance during cellular dehydration. When water is removed from a cell, the formation of extracellular and intracellular ice crystals is reduced with the use of permeating CPAs^19^, decreasing freezing-related injuries. In regard to toxicity, DMSO has been known to be harmful to cells when used in high concentrations and with increasing temperature (0 °C–5 °C is considered to be non-toxic; 20. MeOH, according to both Tsai *et al.*^9^ and Horvath^21^, is less toxic than DMSO. Nevertheless, MeOH is more permeable than DMSO and has been shown to have similar harmful effects to certain organisms^22^. High permeability would also protect the cell more effectively^23^ and when used in moderation, it would benefit the dinoflagellate samples. This could be the reason why higher concentrations of MeOH and DMSO in the present experiment showed lower post-thaw viability than lower concentrations of MeOH and DMSO.

The suitability of a CPA varies according to the samples cryopreserved^20^. In this experiment, MeOH exhibited higher level of protection than DMSO. This may be attributed to the stronger penetrating properties of MeOH which allowed cells to achieve osmotic balance more quickly within the holding time during freezing. Because DMSO is highly toxic at room temperature, there was also the possibility that during the equilibration period and thawing, algae were adversely affected by DMSO. Still the toxicity of DMSO was not apparent in this study because algal viability was maintained between 30%–40%. As for the less effective protection by glycerol, one possible explanation would be high molecular weight and low permeability of the glycerol; thus, it was unable to rapidly replace water within the dinoflagellates during the freezing process^20^,^24^.

The procedural steps of cryopreservation have been known to affect the viability of cryopreserved samples. The holding time is the period in which the samples are cooled by the LN_2_ vapour before plunging into LN_2_. The function of the holding time is to ensure that CPAs replace the intracellular water gradually for preventing a cold shock from the sudden temperature change and intense dehydration when water moves out because of the osmotic change during freezing^25^. The optimal holding time in this experiment was 30 min, whereas in the two-step protocol by Santiago-Vázquez *et al.*^6^, the holding time was two hours at −70 °C using MeOH as the CPA. The difference in holding time between our study and Santiago-Vazquez *et al.*’s^6^ could be attributed to the addition of sucrose, which was not used in the latters’ cryopreservation protocol. The addition of sucrose may promote better cell dehydration and effectively decrease intracellular ice formation in a shorter period of time^26^. Moreover, the addition of sucrose could affect post-thaw viability^27^,^28^. By interacting with the polar heads of the phospholipids, sucrose may have stabilised the membrane when ice crystals grew^29^. This prevented the disruption of cell membrane integrity and improved the survivability of the cryosample. However, the effect of sucrose may be dependent on the type of CPA it is combined with^30^ as the combination of MeOH with sucrose produced better results than the combination of DMSO with sucrose.

The differences in incubation times and temperatures (30 min at room temperature in our study and 10 min at 4 °C in Santiago-Vazquez *et al.*^6^ may have also contributed to the differences in algal viability observed herein versus those documented by Santiago-Vazquez *et al.*^6^. The equilibration period has also been reported to affect viability, as longer equilibration times would mean extended exposure to CPA; hence, CPA toxicity could become an issue, though this depends on not only the types of CPA and its/their concentration(s) but also the sensitivity of the target species^31^. Equilibration temperatures could also influence viability. In algal cryopreservation, the CPAs DMSO and glycerol generally require longer equilibration period (>30 min) to infiltrate cells while MeOH requires less than 30 min^32^. However, equilibration times have ranged widely in past studies of algal cryopreservation: 2–3 min for *Haslea ostrearia*^33^, 5 min for *Porphyra seriata*^34^, and 20 min for *Chlorella vulgaris*, *Isochrysis galbana*, and *Dunaliella salina*^35^. Despite the shorter equilibration used by Santiago-Vazquez *et al.*^6^, it was potentially compensated for during the initial slow-cooling period, as the holding time was 2 hr.

Results showed that ATP concentration was significantly higher in the samples that were kept 5 cm above the LN_2_ surface. However, according to Zhang *et al.*^37^ and Jo *et al.*^10^, slow cooling at the rate of −1 °C per min provided more favourable conditions for the cryopreservation of macroalgae because it allowed adequate time for cells to achieve equilibrium High cooling rate would reduce the period in which cryoprotectant could replace the water, thus diminishing the protective effect of the CPA^37^. The cooling rate applied in this study is similar to those used in seabass sperm cryopreservation studies which required higher freezing rates of > 50 °C per min^38^. Thus, optimum cooling rates are species and cell-type dependent. Even a slight difference in the distance from LN_2_ could affect the cooling rate and consequently, the viability of the sample. Nevertheless, Zhang *et al.*^37^ noted that there was no significant interaction between cooling rates and holding time, although the combination of an optimal cooling rate with the suitable holding time may improve the results.

The cell density studies were consistent with those of Bui *et al.*^39^ and Piasecki *et al.*^40^ which found that higher cell concentrations decreased the cryopreservation efficiency. The reason proposed for this was that hazardous chemical compounds were released from cell walls when cells died. It was found that when algae were frozen, these substances were released in large amounts and that the release could be halted by heat^40^. The inverse relationship between cell density and viability may also be due, in part, to reductions in cell-cell contact, as well as the enhanced likelihood of salt and/or cryoprotectants to penetrate cells when they are at lower densities. Although cryopreserving with 0.64 × 10^6 ^cells/mL yield more viable cells compare to 1.5 × 10^6 ^cells/mL, there difference is insignificant between these two concentration, and it is not practical to cryopreserve lower densities because the main goal is the mass preservation of algae and lower densities would comparably decrease the resulting culturable cells, although cells were more robust at lower concentrations. Thus, 1.5 × 10^6 ^cells/mL would be ideal for freezing studies and were used in subsequent experiments.

In past studies of algae cryopreservation, various viability tests were conducted. For Santiago-Vazqeuez *et al.*’s study^6^, cells were first dyed with trypan blue and then counted under light microscopy. However, Altman *et al.*^41^ had reported that trypan blue can overestimate the amount of viable cells. Consequently, Santiago-Vazqeuez *et al.*’s study^6^ used CellTiter Blue for their culture of post-thawed dinoflagellates. Trypan blue focused on staining dead cells with damaged membrane while CellTiter Blue and ATP bioassay focused on assessing cell metabolic activity. The metabolic activity of a cell provides a more accurate assay of viability when compared with the assessment of membrane integrity because dead cells do not necessarily have ruptured membranes. To the best of our knowledge, the results showed here were the first to use ATP bioassays to assess the viability of cryopreserved marine algae. Authors such as Tsai *et al.*^9^,^14^ have used similar tests on the post-thaw viability of the coral oocytes and sperm sacs. The ATP bioassay quantifies ATP by light emitted from the reaction between luciferin, luciferase and ATP, correlating ATP concentration to the number of cells^42^. Apart from its accuracy in quantification of ATP, it also allows for a straightforward and quick assessment of the viability^43^ with small sample volumes. Although the use of ATP as an indicator of cell health yielded some interesting findings herein, it represents only a snapshot of cell condition. An ATP assay was chosen not only because of its physiological relevance in terms of its role in cellular energy budget/metabolism, but also because of its simplicity and rapidity of assessment. We undertook numerous experiments simultaneously (e.g., cryoprotectant toxicity, effect of distance from liquid nitrogen interface) at relatively narrow time intervals (i.e., 10 minutes), so we needed an assay that could be conducted in a short time-span given the large number of variables (both experimental and physiological) being measured in one experiment. Ultimately, the cell survival data presented are the true indicator of cell viability. It is possible that some cells could be healthy yet have low ATP levels. The ATP assay was an important first step. In future studies, in addition to ATP conduct and cell survival, we aim to explore other cellular and physiological parameters in terms of their efficacy of predicting ultimate cell health and fate (e.g., gene and protein expression, cell culture potential, etc.).

Regardless of the viability outcome of the post-thawed algae, the reproductive ability of the algae remained as the ultimate goal when determining the successful cryopreservation^12^. This is because post-thawed cells with high ATP viability may still lose their cell function gradually as a result of stress from extreme temperatures^44^. Algal culture had been consistently applied in a few studies on algal cryopreservation, such as Santiago-Vazquez *et al.*’s^6^, Bui *et al.*’s^39^ and Zhang *et al.*’s^36^, as a primary or secondary assay of algae viability. In our study, both control and post-thawed algae were incubated to look into their re-population ability. It was observed that post-thawed algae had an initial decrease in their cell count after undergoing cryopreservation. Although the dinoflagellate did not proliferate during the culture period, the cell population was stable during the remaining incubation period. Isolated fresh dinoflagellates (control) also suffered a decrease in the beginning of the culture but for a shorter duration compared with post-thawed algae. The results were similar to Santiago-Vazquez *et al.* where their cell numbers decline in the first 8 weeks followed by a plateau in cell count until week 19. The cell death at the beginning of the incubation period experienced by both control and cryopreserved samples may be attributed to incubation parameters such as temperature, light intensity and light cycle. Cell culture protocols for freshly isolated symbiotic algae are still in their infancy as many clades, including *Symbiodinium* clade G, have yet to be cultured in an *in situ* environment^45^. In addition, these dinoflagellates were freshly isolated from the coral and may require a period of time to acclimate to the free-living state. Prolonged necrosis of post-thawed algae during culture may be attributed to the freezing injury during the cryopreservation–thawing process. The improvement of culturing protocols and techniques, such as thawing and removing CPAs, should be the paramount for survivability of post-thawed algae.

Dinoflagellates of *Symbiodinium* clade G were successfully cryopreserved for the first time in this study. The protocol was effective for protecting the isolated *Symbiodinium* clade G during cryopreservation and the ATP bioassay provided a quick and accurate assessment of metabolic and energy status in algae. Although culture conditions have not attained the desirable outcome for cell proliferation, cell populations were stable within the culture. The methods described here could be applied for future studies on symbiotic algae cryopreservation.

## Material and Methods

### Coral collection

*J. fragilis* was collected from the bay of Houwan (21° 56′ N, 120° 56′ E), Ping Tung County, Taiwan. The corals were randomly selected by SCUBA divers and were immediately attached to a rock substrate and transferred into fresh seawater in an upright position. The corals were kept in tanks with natural seawater during the experiments. The collection was permitted by the Kenting National Park Management Office (102.0618.0841).

### Dinoflagellates extraction

The extraction of dinoflagellates was performed according to the following method: the epidermis layer of the *J. fragilis* coral was removed with a scalpel and placed into a petri dish containing filtered seawater (FSW). Seawater used in all experimental procedures was prepared by filtering through a 0.2 μm membrane filter (ADVANTEC, Japan) using a vacuum pump (Rocker 300; Rocker Scientific, Taiwan). With the underside of the epidermis facing upwards, curved tweezers were used to apply pressure onto the surface of the inner epidermis and dinoflagellates were collected using pipette suction. The aggregating dinoflagellates were separated through a 23G × 1 inch syringe needle. The coral tissues that precipitated at the bottom of the tube were removed. Then, the samples were centrifuged (Microcentrifuge 5417R; Eppendorf, Germany) three times at 300 x *g* for 3 min at 25 °C with filtered seawater for removing the remaining coral cells and tissues. Aliquots of 10 μL of the samples were then pipetted into Neubauer counting chambers for determining the density of the dinoflagellates in the samples. The total count of each area was averaged and the density of the sample was diluted to approximately 1.0 × 10^6 ^cells/mL.

### Two-Step freezing

**Figure 6:**
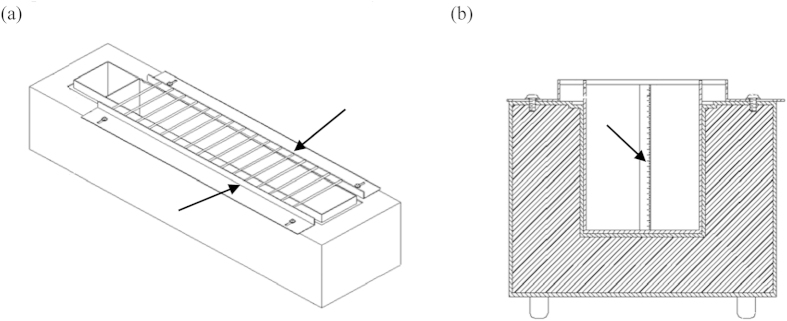
(**a**) Patented vapour device viewed from the top. Indentations (arrows) on top of the device indicate the position of the straw placed for preventing the straw from falling into LN_2._ (**b**) Lateral view of the blue print of the patented vapour device. A measuring ruler (indicated by the arrow) was attached by the side for accurately measuring the height of LN_2_.

CPAs were prepared using FSW in the following concentrations: 1 M and 3 M methanol (MeOH), 2 M and 3 M dimethyl sulfoxide (DMSO), 1 M glycerol (Gly), 2 M ethylene glycol (EG), 2 M propylene glycol (PG) and 0.2 M and 0.4 M sucrose. PG, EG, and Gly were purchased from JT Baker (Phillipsburg, NJ., U.S.A.), DMSO and sucrose were purchased from Sigma (St. Louis, MO., U.S.A.) and MeOH was purchased from Merck (Darmstadt, Germany). Freezing procedures were conducted by adding those CPAs to the sample by pipetting and the mixtures were equilibrated at room temperature for 10, 20 or 30 min. The equilibrated samples were then loaded into straws and suspended 3 cm, 5 cm and 7 cm above the LN_2_ surface for 30 min on the cooling device (Patent no.: M394447; Fig. 6a,b). A digital thermometer (TM-903A, Lutron Electronics, U.S.A) was used to measure the temperature and the cooling rate of LN_2_ at 3 cm, 5 cm and 7 cm from the LN_2_ sample. The straws were then plunged into LN_2_ and immersed for at least 10 min. Each straw was retrieved from the cooling device and immersed in a 37 °C water bath (MajorScience, Taiwan) for 10 sec for thawing. The thawed samples were loaded into the vials for ATP assays.

### Optimum Cell density

Dinoflagellates were also tested at different cell concentrations to determine the optimum concentration for the cell preservation: 0.64 × 10^6^, 1.5 × 10^6^ and 2.5 × 10^6 ^cells/mL. The cell density experiment was conducted in triplicates using the final freezing protocol that was established (30 min holding time, 5 cm from LN_2_ and with 1 M methanol and 0.4 M sucrose). The viability was only assessed with the ATP bioassay.

### Viability of algae

Intracellular ATP concentration was used to determine the viability of algae that had undergone chilling and freezing with an ApoSENSOR™ ATP Cell Viability Bioluminescence Assay Kit (BioVision, U.S.A). For each sample, an aliquot of 10 μL of the thawed sample was transferred into a vial supplied with the kit and 100 μL of Nucleotide Releasing Buffer was added. The mixture was incubated for 3 min before the addition of 5 μL of ATP Monitoring Enzyme. The ATP concentration was measured using the light produced when the sample reacted with firefly luciferase using a Lumat 9507 luminometer (Berthold Technologies, Wildbad, Germany). The amount of light produced was directly proportional to the amount of ATP present in the sample. Dinoflagellates were then cultured, further assessing the survivability.

### Algae Culture

The thawed dinoflagellates were centrifuged (300× *g*, 5 min, 25 °C) for removing the CPA. Fresh FSW was added for resuspending the samples. The samples were then transferred into a 24-well microplate (Cosmo Biosciences Inc., CA, U.S.A.) which was sealed and stored in a culture incubator set to a 12 h light:12 h dark cycle (Kansin Instruments CO., LTD.). The culture medium was prepared by mixing 32 g of Coralife Salt Water Mix (WI, U.S.A) with 1 L of distilled water to create 32 ppt artificial seawater. After the Salt Water Mix was fully dissolved, 30 mL of Guillard’s f/2 medium and 1 mL of an antibiotic solution were added to 970 mL of the artificial seawater. Both the antibiotics (penicillin: 100 units/ml; streptomycin: 10 mg/ml) and Guillard’s f/2 medium were purchased from Sigma-Aldrich (St. Louis, MO., U.S.A.). The solution was filtered using 0.45 μm filter (Thermo Scientific Inc., Massa., U.S.A.) after it had evenly dissolved. The culture was replenished with fresh medium every two days. During the medium exchange, 10 μL aliquot of the culture was retrieved for cell counts.

### Statistical analysis

Triplicates were carried out for each treatment and the measured outcomes were averaged. Normalized oocyte survival was used to assess oocyte viability and was calculated as follows:

Normalized oocyte viability (%) = 100/relative units of light (RUL) of untreated control × experimental RUL (%).

Statistical analyses were carried out using SPSS (Version 17) and Microsoft Excel. First, the one-sample Kolmogorov–Smirnov test was used to ascertain that data were normally distributed. One-way ANOVAs and LSD test were used to analyse statistical significance of each treatment. Data were expressed as mean ± SEM and *p* values of less than 0.05 were considered to be statistically significant.

## Additional Information

**How to cite this article**: Chong, G. *et al.* Cryopreservation of the gorgonian endosymbiont Symbiodinium. *Sci. Rep.* **6**, 18816; doi: 10.1038/srep18816 (2016).

## Figures and Tables

**Table 1:** Effect of distance to surface of LN_2_ on final temperatures and freezing rates.

Distance(cm)	Initial Temperature(°C)	Final Temperature(°C)	Temperature change(°C)	Time Interval(min)	Freezing rate(°C/min)
3	23.8	−172.2	−196.0	3.35	−58.51
5	24.5	−123.9	−148.4	2.48	−59.83
7	24.0	−112.9	−136.9	3.28	−41.70
